# Case Report: How to tailor adjuvant radiotherapy after open partial laryngeal surgery in the setting of a secure R0-resection?

**DOI:** 10.3389/fonc.2026.1879804

**Published:** 2026-08-27

**Authors:** Pierluigi Bonomo, Maja Guberina, Jon Cacicedo

**Affiliations:** 1 Radiation Oncology, Azienda Ospedaliero-Universitaria Careggi, Florence, Italy; 2 Clinic and Polyclinic for Radiotherapy and Radiation Oncology, Experimental Radiation Biology, University Hospital Essen, Essen, Germany; 3 National Center for Tumor Diseases West, Essen, Germany; 4 Department of Radiation Oncology, Cruces University Hospital/University of the Basque Country, Barakaldo, Spain

**Keywords:** dose-guided optimization, functional outcome, image guided radiotherapy (IGRT), multidisciplinary approach, open partial horizontal laryngectomy (OPHL), optimized treatment outcome, radiotherapy treatment planning, supraglottic cancer

## Abstract

In this report, we discuss this clinical case, when the laryngeal remnant, or “neo-larynx”, is not part of the clinical target volume, it should be delineated as a bona-fide organ at risk or avoidance structure: recognizing the post-surgical anatomy could help maximize the preservation of the functional outcome. In addition, raising awareness on this rare yet very challenging disease presentation could foster a more homogeneous practice among radiation oncologists treating laryngeal cancer analogue to thoracic cancer.

## Clinical scenario

**Figure 1:**
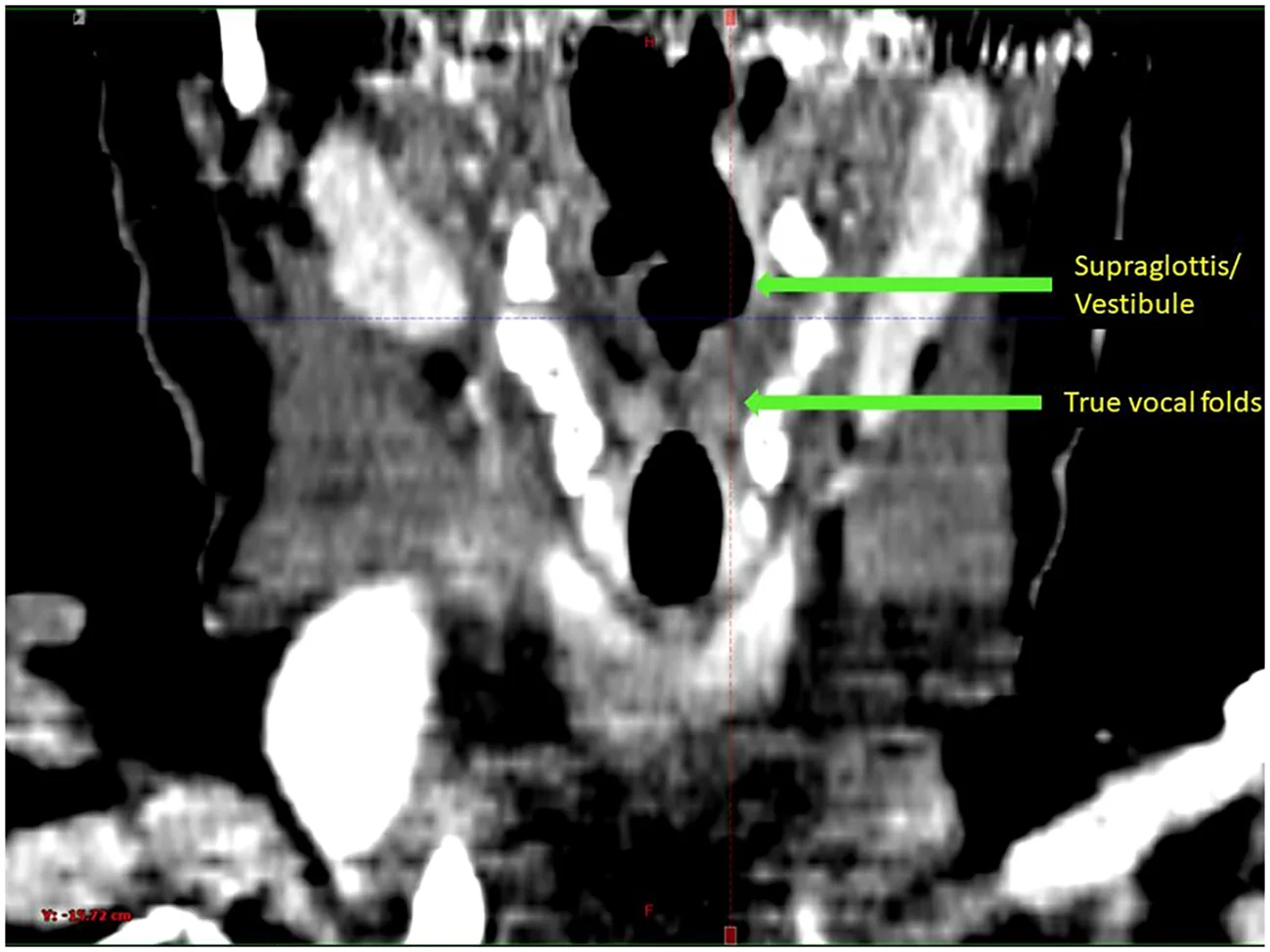
Supraglottis/Vestibule: The aryepiglottic fold forms a muscular and mucosal rim at the entrance of the larynx, continuing inferiorly into the ipsilateral false vocal cord. .

A 61-year-old woman presented with mild left ear numbness and dysphonia, which had worsened over a three-month period. After proper work-up, she was diagnosed with squamous cell carcinoma of the supraglottic larynx, staged as cT2 cN0 cM0. On fiberoptic examination, an ulcerated lesion extended from the laryngeal surface of the epiglottis to the left aryepiglottic fold and the ipsilateral false vocal cord. The aryepiglottic fold forms a muscular and mucosal rim at the entrance of the larynx, continuing inferiorly into the ipsilateral false vocal cord (**Figure 1**). The vocal cord mobility was unaffected. The patient did not complain of dysphagia.

Additional preoperative radiological imaging, together with endoscopic examination, allowed definition of the anatomical borders and characteristics of the lesion and guided further surgical planning. The patient was offered organ-preservation surgery. Open partial horizontal laryngectomy (OPHL type 1) with bilateral selective neck dissection was performed (1–4). The patient did not require a tracheostomy.

The pathologic report documented minimal focal invasion of the pre-epiglottic space < 0.5 mm, whereas no perineural or lymphovascular invasion were noted. Unexpectedly, a total of eight lymph nodes were positive, four on each side. In addition, three of the four positive left-sided lymph nodes demonstrated microscopic extranodal extension. Thus, the pathologic findings were consistent with pT3 pN3b R0 pL0 pPn0 pV0. For the primary tumor, an R0 resection was achieved, with no microscopic residual disease, and all mucosal resection margins in tumor areas not surrounded by cartilage were greater than 5 mm.

**Figure 2:**
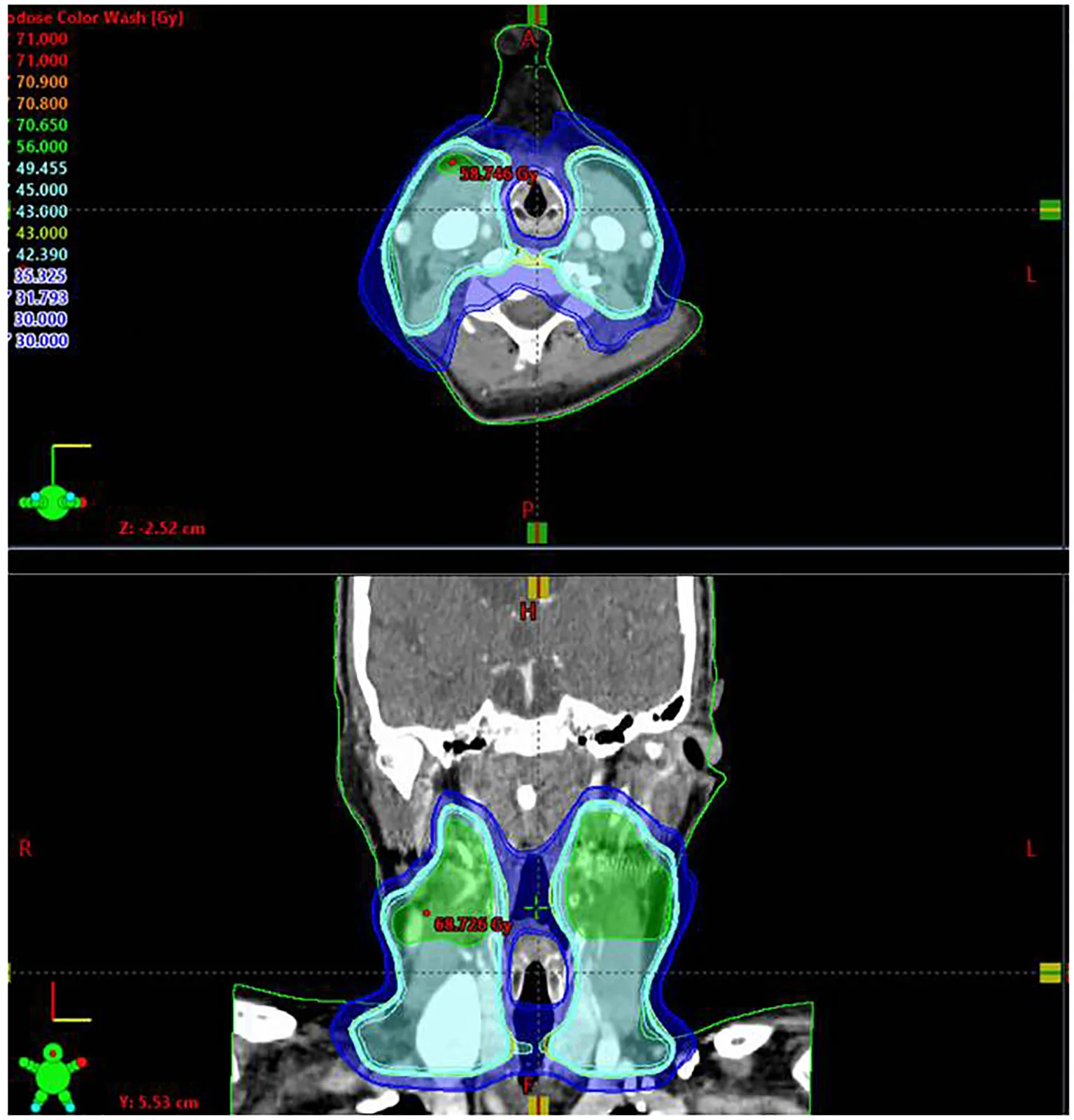
External Beam Planning: The neo-larynx may be delineated as a *bona fide* organ at risk (OAR) with the objective of preserving phonatory, swallowing, and airway functions.

**Figure 3:**
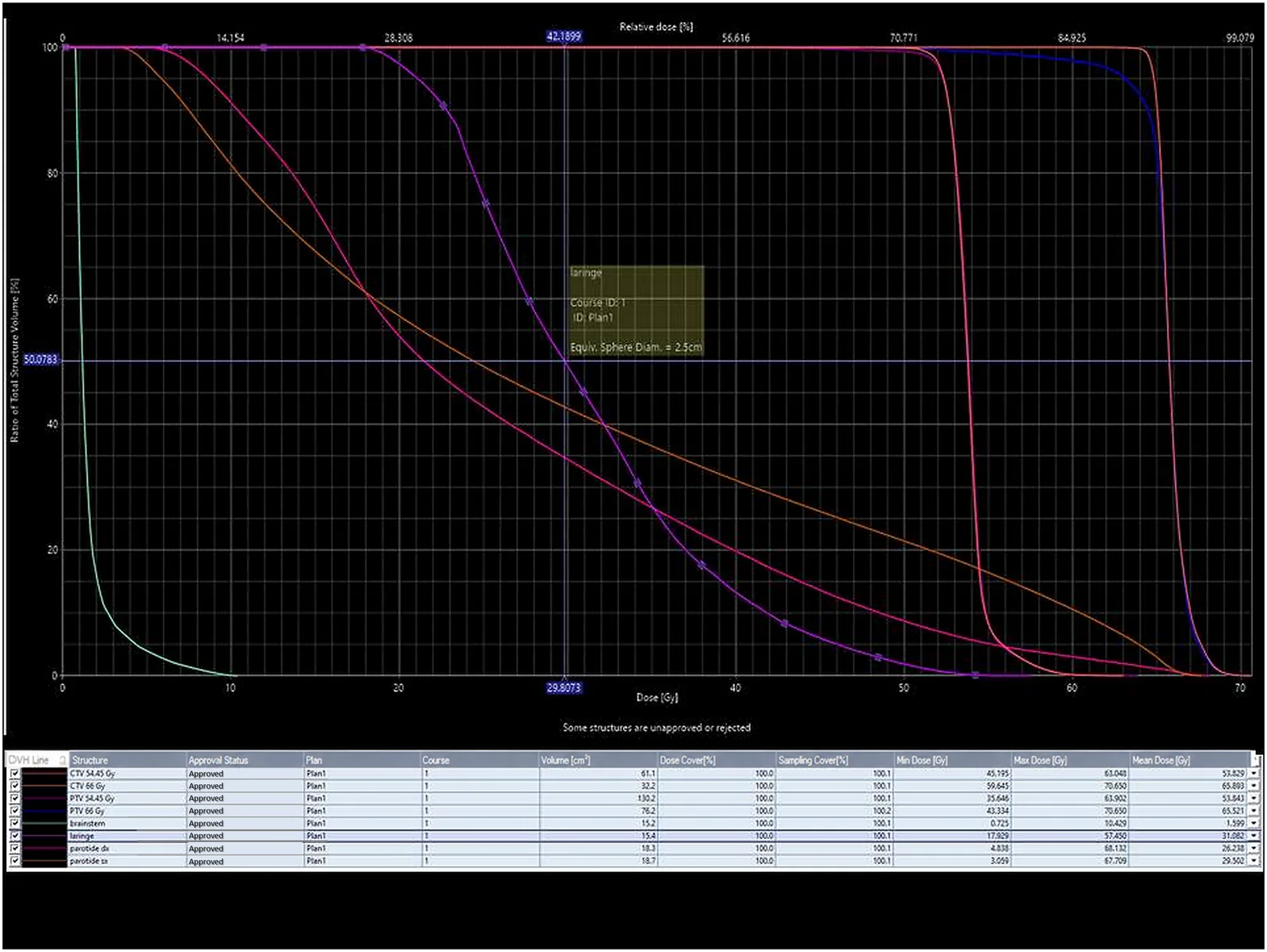
External Beam Planning: dose volume histogram.

**Figure 4:**
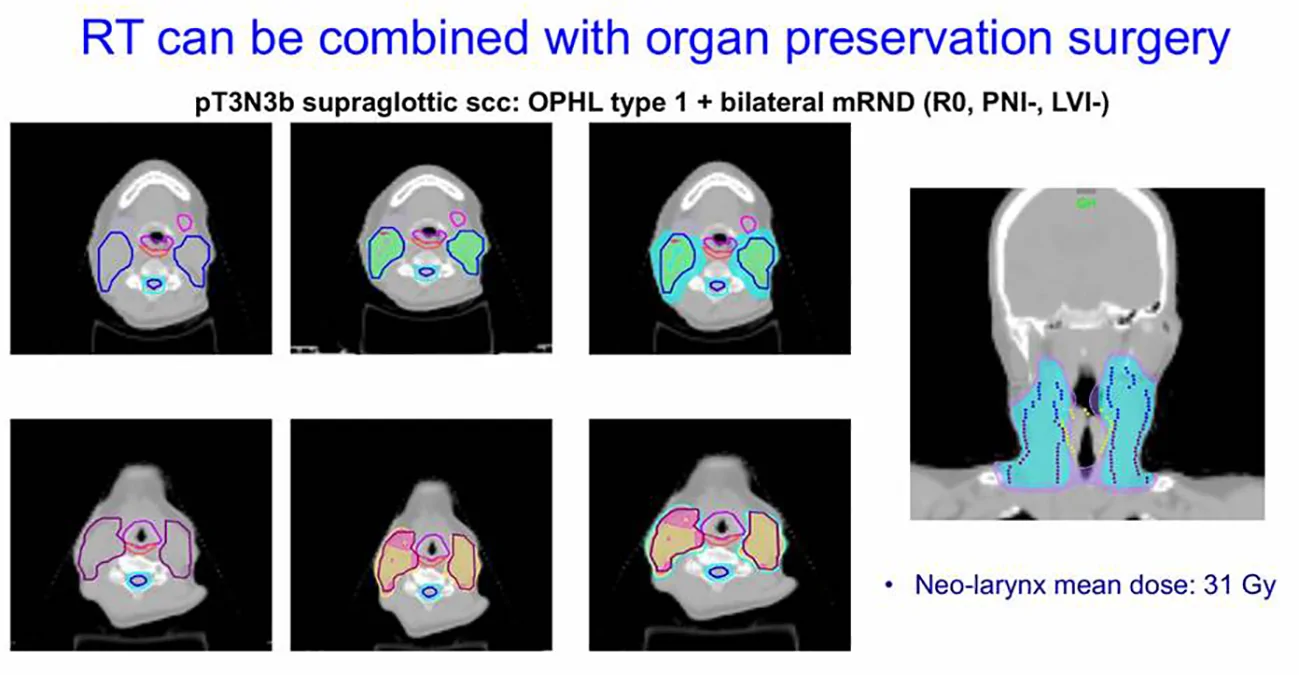
Overall treatment plan and dose distribution. After open partial horizontal laryngectomy (OPHL), the postsurgical “neo-larynx” was regarded as an organ at risk, particularly because the pathologic margins were negative and there was no direct evidence of residual disease involving the reconstructed laryngeal remnant.

In light of the pathologic report, the initial indication for single-modality treatment with no anticipated need for adjuvant therapy was revised because of the high-risk pathologic findings. The patient proceeded to adjuvant radiotherapy in line with the most recent consensus recommendations for laryngeal cancer (statement 35) (2) and the NCCN Guidelines (3). The treatment was performed with image-guided radiotherapy. The patient proceeded to adjuvant radiotherapy. Using a simultaneous integrated boost approach, 66 Gy and 54.45 Gy were delivered in 33 fractions to the dissected ENE-positive and ENE-negative lymph node levels, respectively (**Figures 1**–**4**). In line with the most recent consensus recommendations for laryngeal cancer (statement 35) (2), the laryngeal remnant was intentionally spared because of the absence of major risk factors and received a mean dose of 31 Gy. No concomitant chemotherapy was administered because of significant comorbidities (cardiac comorbidity, liver cirrhosis), and patient's informed choice.

Postoperatively and after radiotherapy, the patient demonstrated a very good functional recovery. Oral intake was resumed without clinically significant dysphagia (CTC AE grade 0), and swallowing function remained normal throughout follow-up, with no evidence of aspiration or dietary restriction. Speech was preserved, with normal articulation and intelligibility, and the patient reported no speech-related functional limitations. The following postoperative scores are recorded: Functional Oral Intake Scale (FOIS) (4), score of 7; Speech Intelligibility Rating (SIR), score of 2; and M. D. Anderson Dysphagia Inventory (MDADI) (5), score of 80. After 2 years, the patient remained disease-free, with no late adverse effects.

Adjuvant radiotherapy to the laryngeal remnant is generally indicated after OPHL in the presence of major risk factors, such as pT4a, close or positive margins, and positive PNI and/or LVI1. In these cases, it is generally agreed that adjuvant treatment may increase the risk of long-term functional complications, such as permanent tracheostomy; a total dose of no more than 56 Gy has been suggested to minimize the risks. Although the literature is limited and no high-level of evidence is available, a recent meta-analysis (6) of 1,959 patients treated with open partial surgery, most of whom underwent OPHL type 1, did not demonstrate a clear detrimental effect of postoperative radiotherapy.

In the adjuvant setting, delivering dose levels tailored to the differential microscopic risk profile is an established cornerstone of modern intensity-modulated radiation therapy (IMRT). It is noteworthy that, for one of the potentially most hazardous situations, namely postoperative radiotherapy after open partial laryngeal surgery, no guidance is available for practice radiotherapy treatment planning in clinical practice.

In this report, we discuss this clinical case and propose that, when the laryngeal remnant, or “neo-larynx,” is not part of the clinical target volume, it should be delineated as a bona-fide organ at risk or avoidance structure. Recognizing the postsurgical anatomy could help maximize functional preservation. In addition, raising awareness on this rare yet highly challenging clinical presentation could promote a more homogeneous approach among radiation oncologists treating laryngeal cancer, analogous to that in thoracic oncology (7). Written informed consent was obtained from the patient, and ethics approval was granted by the University of Florence.

## Focus section

1. How should the laryngeal remnant, or “neo-larynx,” be considered when it is not part of the clinical target volume and is therefore delineated as a *bona fide* organ at risk, including dose limitations? What contouring considerations are important for recognizing the postsurgical anatomy to maximize structural preservation and optimize functional outcomes?
2. What are the essential technical prerequisites and treatment-planning considerations for standardizing clinical practice among radiation oncologists, including the creation of an avoidance structure in the RT plan?
3. Discuss the risk–benefit ratio of intentionally constraining the dose to the laryngeal remnant after OPHL to personalize treatment for patients with laryngeal cancer undergoing upfront organ-preservation surgery.

To Question 1: After open partial horizontal laryngectomy (OPHL), the postsurgical “neo-larynx” could be regarded as an organ at risk, particularly when pathologic margins are negative and there is no direct evidence of residual disease involving the reconstructed laryngeal remnant. The neo-larynx may be delineated as a *bona fide* organ at risk (OAR) with the objective of preserving phonatory, swallowing, and airway functions (**Figures 2**–**4**).

A careful understanding of the altered anatomy after OPHL is therefore mandatory. Radiation oncologists should work closely with the surgeon and pathologist and recognize the remaining functional structures, including the cricoid cartilage, arytenoid(s), cricoarytenoid unit(s), reconstructed tongue base, hyoid remnants, and the mucosal neolaryngeal lumen. Delineation should rely on operative reports and postoperative CT/MRI fusion whenever available. Endoscopic correlation and even mapping are highly valuable, analogous to their use in thoracic oncology (6–9). Furthermore, it may be useful to identify the residual vibratory and sphincteric components that are crucial for functional recovery.

The contouring strategy should aim to exclude the neolarynx from elective high-dose irradiation whenever oncologically acceptable. Particular attention should be paid to preserving at least one functional cricoarytenoid unit, as this structure is associated with swallowing competence and decannulation. Dose limitation to the neo-larynx may reduce edema, fibrosis, chondronecrosis, dysphagia, aspiration, and late airway compromise. Functional substructures such as the arytenoid remnant, cricoid cartilage, and reconstructed neolarynx should therefore be systematically identified and spared if feasible.

To Question 2: To standardize practice among radiation oncologists, several technical and methodological prerequisites are required.

First, a dedicated contouring protocol for post-OPHL anatomy should be established. This includes consensus-based atlases defining the neo-larynx and its substructures as avoidance structures distinct from the tumor-bed CTV. Multidisciplinary collaboration between surgeons, radiation oncologists, and radiologists is essential to correctly interpret postoperative anatomy (10).

Second, high-quality treatment-planning imaging is mandatory. Thin-slice planning CT with intravenous contrast should be complemented by MRI and/or fusion with preoperative imaging whenever feasible. Rigid or deformable image registration can improve the identification of residual functional anatomy and areas at genuine risk of microscopic disease.

Third, intensity-modulated radiotherapy (IMRT) or volumetric-modulated arc therapy (VMAT) should be considered essential techniques for achieving highly conformal dose distributions. Adaptive radiotherapy is under evaluation (11). These technologies allow the creation of a dedicated avoidance structure corresponding to the neo-larynx, enabling intentional dose de-escalation while maintaining adequate target coverage.

Planning considerations could include: (i) explicit delineation of the neo-larynx as an OAR; (ii) separation of the high-risk postoperative bed and the functional laryngeal remnant; (iii) robust image-guided radiotherapy (IGRT) protocols to reduce setup uncertainty (11); and (iv) adaptive replanning in cases of significant anatomical changes during treatment (11).

To Question 3: The concept of intentionally constraining radiation dose to the laryngeal remnant after OPHL reflects a paradigm shift toward functional organ preservation rather than merely anatomical preservation. The principal benefit of this strategy is the potential reduction in long-term functional toxicity, including dysphagia, aspiration, chronic edema, impaired voice quality, tracheostomy dependence, and tissue necrosis.

Patients undergoing upfront organ-preservation surgery already possess a compromised but functional neo-larynx. Delivering unrestricted postoperative radiation doses may jeopardize the delicate functional equilibrium achieved surgically. Therefore, selective dose reduction to the residual laryngeal structures could improve quality of life, swallowing rehabilitation, and social reintegration.

However, this strategy must be balanced against the oncologic risk of undertreatment. Inadequate dose coverage of areas at genuine risk of microscopic residual disease could theoretically increase local recurrence rates. Consequently, patient selection is critical.

In our patient, we observed a very good long-term outcome without significant functional impairment.

The therapeutic challenge is therefore to personalize postoperative radiotherapy according to both oncologic and functional risk profiles. Modern IMRT/VMAT and ART techniques (10, 11), improved contouring standardization, and multidisciplinary decision-making (12) enable a more individualized balance between tumor control probability and normal tissue complication probability.

Future prospective studies correlating delivered dose to neo-laryngeal substructures with functional outcomes and local control are needed to establish validated dose constraints and optimize patient selection for this personalized approach.

## Data Availability

The datasets presented in this article are readily available upon request. Requests to access the datasets should be directed to bonomop@aou-careggi.toscana.it.

## References

[1] Meliante PG Battilocchi L Costantino A Lee K Moon SJ Ralli M . Transoral robotic vertical partial laryngectomy (hemilaryngectomy) extended to the hypopharynx. Head Neck. (2024) 46:708–12. doi: 10.1002/hed.27634 38221740

[2] Ferrari M Mularoni F Smussi D Gaudioso P Bonomo P Friborg J . International consensus on laryngeal preservation strategies in laryngeal and hypopharyngeal cancer. Lancet Oncol. (2025) 26:e264–81. doi: 10.1016/S1470-2045(25)00020-8 40318658

[3] National Comprehensive Cancer Network . N CCN Clinical Practice Guidelines in Oncology (NCCN Guidelines®): Head and Neck Cancers (Version 2.2026. (2026) 102. Available online at: https://www.nccn.org/guidelines/guidelines-detail?category=1&id=1437 (Accessed June 30, 2026).

[4] Hamzic S Braun T Juenemann M Butz M Voswinckel R Belly M . Validation of the german version of functional oral intake scale (FOIS-G) for flexible endoscopic evaluation of swallowing (FEES). Dysphagia. (2021) 36:130–9. doi: 10.1007/s00455-020-10114-1 32342178 PMC7803872

[5] Chen AY Frankowski R Bishop-Leone J Hebert T Leyk S Lewin J . The development and validation of a dysphagia-specific quality-of-life questionnaire for patients with head and neck cancer: the M. D. Anderson dysphagia inventory. Arch Otolaryngol Head Neck Surg. (2001) 127:870–6. 11448365

[6] Locatello LG Jiang S Chen L Caini S Maggiore G Dong P . Oncological and functional impact of adjuvant treatments after open partial laryngeal surgery: a systematic review of the literature and a meta-analysis. Eur Arch Otorhinolaryngol. (2023) 280:2911–26. doi: 10.1007/s00405-023-07871-8 36806990 PMC10175366

[7] Guberina M Herrmann K Pöttgen C Guberina N Hautzel H Gauler T . Prediction of Malignant lymph-nodes in stage III NSCLC by machine-learning classifiers using EBUS-TBNA and [18F]FDG-PET/CT. In: Sci Rep. London, Berlin: Springer Nature (2022). doi: 10.1038/s41598-022-21637-y PMC9584941 36266403

[8] Guberina M Darwiche K Hautzel H Pöttgen C Guberina N Gauler T . Patterns of nodal spread in stage III NSCLC: importance of EBUS-TBNA and 18F-FDG PET/CT for radiotherapy target volume definition. Radiat Oncol. (2021) 16:176. doi: 10.1186/s13014-021-01904-4 34526050 PMC8442338

[9] Guberina M Darwiche K Hautzel H Ploenes T Pöttgen C Guberina N . Impact of EBUS-TBNA in addition to 18F-FDG PET/CT imaging on target volume definition for radiochemotherapy in stage III NSCLC. Eur J Nucl Med Mol Imaging. (2021) 48(9):2894–903. doi: 10.1007/s00259-021-05204-7 33547554 PMC8263445

[10] Kürten CHL Zioga E Gauler T Stuschke M Guberina M Ludwig JM . Patterns of cervical lymph node metastasis in supraglottic laryngeal cancer and therapeutic implications of surgical staging of the neck. Eur Arch Otorhinolaryngol. (2021) 278:5021–7. doi: 10.1007/s00405-021-06753-1 33772318 PMC8553708

[11] Guberina M Guberina N Hoffmann C Gogishvili A Freisleben F Herz A . Prospects for online adaptive radiation therapy (ART) for head and neck cancer. Radiat Oncol. (2024) 19:4. doi: 10.1186/s13014-023-02390-6 38191400 PMC10775598

[12] Baehr A Guberina M Ernst P Koch K Juretko M Nestle U . Current practices in peer review in German radiation oncology: a nationwide survey. Strahlenther Onkol. (2025) 202:496–507. doi: 10.1007/s00066-025-02444-6 40705058 PMC13109161

